# Prognostic value of a 92-probe signature in breast cancer

**DOI:** 10.18632/oncotarget.3525

**Published:** 2015-04-11

**Authors:** Salima Akter, Tae Gyu Choi, Minh Nam Nguyen, Abel Matondo, Jin-Hwan Kim, Yong Hwa Jo, Ara Jo, Muhammad Shahid, Dae Young Jun, Ji Youn Yoo, Ngoc Ngo Yen Nguyen, Seong-Wook Seo, Liaquat Ali, Ju-Seog Lee, Kyung-Sik Yoon, Wonchae Choe, Insug Kang, Joohun Ha, Jayoung Kim, Sung Soo Kim

**Affiliations:** ^1^ Department of Biochemistry and Molecular Biology, School of Medicine, Kyung Hee University, Seoul, Republic of Korea; ^2^ Department of Biochemistry and Cell Biology, Bangladesh University of Health Sciences, Dhaka, Bangladesh; ^3^ Department of Systems Biology, The University of Texas MD Anderson Cancer Center, Houston, Tx, USA; ^4^ Departments of Surgery and Biomedical Sciences, Samuel Oschin Comprehensive Cancer Institute, Cedars-Sinai Medical Center, Los Angeles, CA, USA

**Keywords:** microarray, gene signature, breast cancer, prognosis

## Abstract

Clinical applications of gene expression signatures in breast cancer prognosis still remain limited due to poor predictive strength of single training datasets and appropriate invariable platforms. We proposed a gene expression signature by reducing baseline differences and analyzing common probes among three recent Affymetrix U133 plus 2 microarray data sets. Using a newly developed supervised method, a 92-probe signature found in this study was associated with overall survival. It was robustly validated in four independent data sets and then repeated on three subgroups by incorporating 17 breast cancer microarray datasets. The signature was an independent predictor of patients' survival in univariate analysis [(HR) 1.927, 95% CI (1.237–3.002); *p* < 0.01] as well as multivariate analysis after adjustment of clinical variables [(HR) 7.125, 95% CI (2.462–20.618); *p* < 0.001]. Consistent predictive performance was found in different multivariate models in increased patient population (*p* = 0.002). The survival signature predicted a late metastatic feature through 5-year disease free survival (*p* = 0.006). We identified subtypes within the lymph node positive (*p* < 0.001) and ER positive (*p* = 0.01) patients that best reflected the invasive breast cancer biology. In conclusion using the Common Probe Approach, we present a novel prognostic signature as a predictor in breast cancer late recurrences.

## INTRODUCTION

Breast cancer is the leading cause of cancer-related deaths amongst women worldwide [1] and it is recognized to be a molecularly heterogeneous disease [2]. DNA microarray technology has the potential to identify breast cancer gene signatures which can improve diagnosis and risk stratification [3–5]. Most gene expression profiling studies, however, have been performed on relatively small data sets resulting in overfitting of the training [2, 4, 6]. To develop a stable signature in such a profiling, at least thousand samples are needed [7]. Meta-analysis is considered to be a promising approach to overcome this limitation by combination of microarray data sets [8]. However, this might have some common problems such as challenges of different probes in individual microarray chips with varying in precision, different relative scales, and diverse dynamic ranges [9, 10]. It has also been shown that robust identification of prognostic signature is performed either by the combination of identical [11] or different microarray chip [12]. In both approaches, confined probe sets are used because the former method incorporates limited number of probes while the latter excluded majority of the genes required to predict the patient's outcome. In different combinations, matched probe identification numbers (ID) or gene symbols may further increase measurement bias [10]. Lack of independent and/or additional validation may also lead to uncertainty of the prognostic signature in clinical application. There is thus a need to identify a prognostic signature that would solve the problems of small patient data and also preserve the predictive strength without combining microarray data, to accurately predict the patient's outcome as well as the biology of the disease.

In the present study, we identified a novel probe signature by reducing baseline differences, incorporating a large number of probes and patients (677), and updated primary breast cancer datasets to improve predictor performance. In addition, we tested whether the identified gene signature could robustly validate in independent and combined data sets. Finally, we attempted to demonstrate whether this signature could distinguish subtypes of breast cancer reflecting the biological and clinical characteristics of the disease.

## RESULTS

### Identification of common probe sets

We selected three recent microarray data of primary human breast cancer considering both high and moderate quality of gene expression, cancer cells content (>60%), patients number (*n* > 100), treatment regimen (2/3^rd^ untreated before surgery) and previous survival association. Detailed information and distribution of several clinical variables for these data sets are shown in Tables 1 and 2. A flow chart showing the identification of common probe sets is depicted in Supplementary Figure S1. Each of the three data sets were filtered individually on the log_2_ scale with at least five observations that represented the same probe expression level. As a result, 810, 1024 and 918 probe sets were identified from data sets 1, 2, and 3, respectively, and were passed through a Venn diagram generator that produced 408 common probe sets. The heatmap of these common probe sets for the three data sets is shown in Supplementary Figure S2.

**Table 1:** Breast cancer microarray datasets used in this study

GEO Number	Origin/Year	Author	Paper Title	Chip type
Data set 1GSE42568	Ireland, 2013	*Clarke et al*	Breast Cancer Gene Expression Analysis	HG-U133_Plus_2
Data set 2GSE20685	Taiwan, 2011	*Kao et al*	Microarray-based molecular subtyping of breast cancer	HG-U133_Plus_2
Data set 3GSE31448	France, 2011	*Sabatier et al*	Down-regulation of ECRG4, a candidate tumor suppressor gene in human breast cancer	HG-U133_Plus_2
Data set 4GSE12276	Netherlands, 2009	*Bos et al*	Expression data from primary breast tumors	HG-U133_Plus_2
Data set 5GSE48390	Taiwan, 2013	*Huang et al*	Concurrent Gene Signatures for Han Chinese Breast Cancers	HG-U133_Plus_2

GSE, GEO datasets number prefixes; HG-U133_ Plus_ 2, a type of oligonucleotide gene chip from the Affymetrix.

**Table 2:** Clinical and demographical characteristics of the patients

Variable	Data set 1	Data set 2	Data set 3	Data set 4	Data set 5
**Number of patients**	104	327	246	195	81
**Median age at diagnosis (years)**	56 (31–90)	46 (24–84)	54.5 (24–84)		
**Median follow-up (months)**	63 (4.6–111)	97 (5–169)	54.2 (3.4–222.3)	27 (3–115)	50 (0.9–69.0)
**Tumor Grade**					
**I**	11 (10.5%)		43 (17.5%)		
**II**	40 (38.5%)		84 (34.1%)		
**III**	53 (51.0%)		119 (47.2%)		
**N/A**			3 (1.2%)		
**Estrogen Receptor**					
**Yes**	67 (64.4%)		139 (56.5%)		53 (65.4%)
**No**	34 (32.7%)		105 (42.7%)		28 (34.6%)
**N/A**	3 (2.9%)		2 (0.8%)		
**Progesterone Receptor**					
**Yes**			120 (48.8%)		
**No**			124 (50.4%)		
**N/A**			2 (0.8%)		
**Lymph Node**					
**Yes**	59 (56.7%)		129 (52.4%)		
**No**	45 (43.3%)		115 (46.8%)		
**N/A**			2 (0.8%)		
**Tumor Size**					
**< 5 cm**	96 (92.3%)				
**> 5 cm**	8 (7.7%)				

Data set 1, Ireland cohorts (GSE42568); Data set 2 and 5, Taiwan cohorts (GSE20568 and GSE48390 respectively); Data set 3, France cohorts (GSE31448); Data set 4, The Netherlands cohorts (GSE12276); N/A, not available.

### Development of a prognostic survival signature and risk prediction

**Figure 1:**
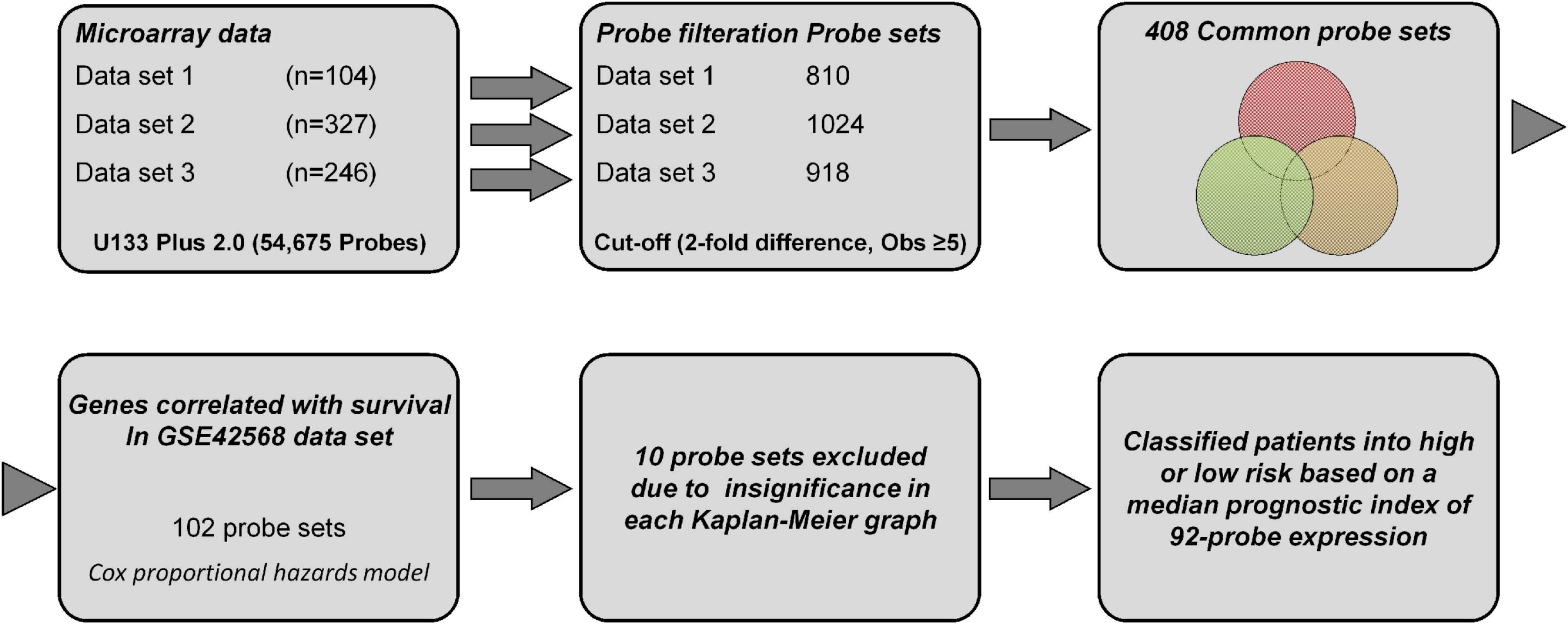
A workflow in this study

**Figure 2:**
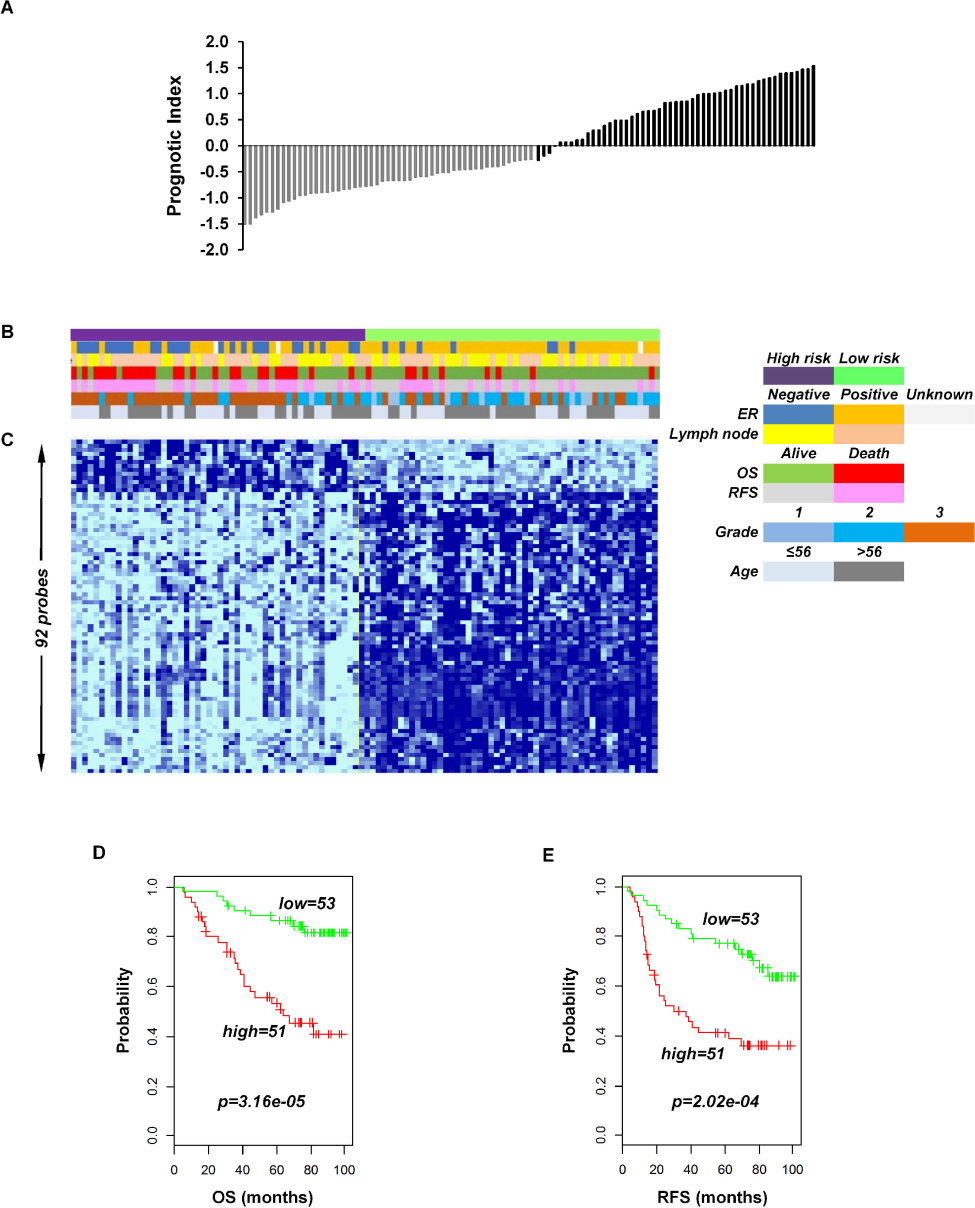
Association of the 92-probe signature in respect to clinical and survival information of 104 primary breast tumor patients in training dataset 1 **A.** Prognostic index in dataset 1. Each bar represents the prognostic index for an individual patient. **B.** The association of survival and clinical information within the two risk groups in dataset 1. **C**. The heatmap of the median-centered 92-probe expression profile (green, relative high expression; sky blue, relative low expression). **D.** and **E.** Kaplan-Meier plots of the two subgroups in the training cohort predicted by CCP. *p* values were obtained from log-rank test. The ‘+’ symbols in the panels indicate censored data. CCP, compound covariate predictor; OS, overall survival; RFS, relapse free survival.

**Figure 3:**
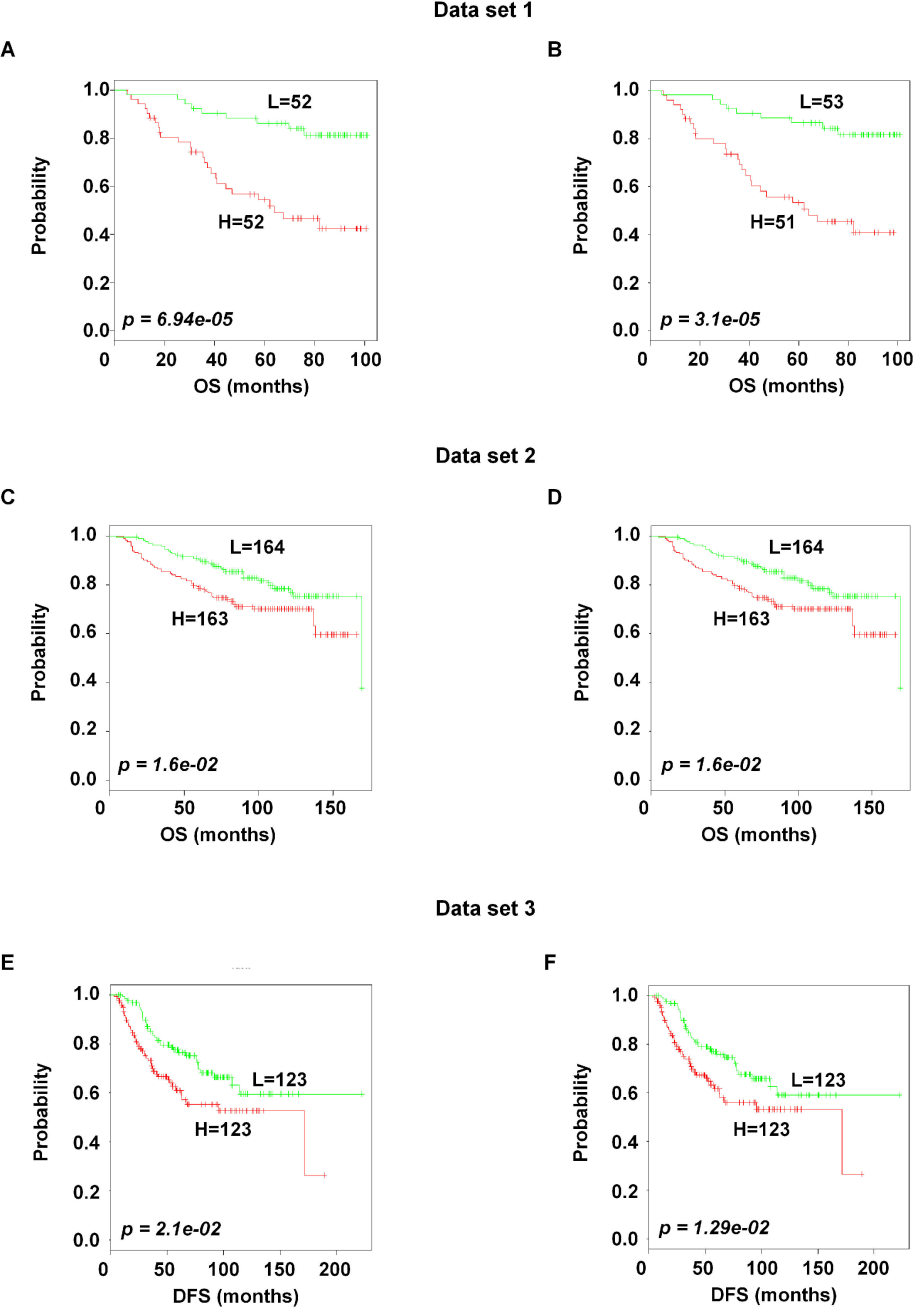
Association of the 102 and the 92-probe sets with survival information of primary breast tumor patients in dataset 1, 2 and 3 respectively **A–F.** Kaplan-Meier plots of the two subgroups were predicted by CCP. (A and B) Dataset 1. (C and D) dataset 2. (E and F) dataset 3. *p* values were obtained from log-rank test. The ‘+’ symbols in the panels indicate censored data. CCP, compound covariate predictor; OS, overall survival; DFS, disease free survival.

To identify the prognostic gene candidates, we used the univariate Cox regression to generate hazard ratio using the Cox regression coefficient of each probe in the prognostic signature. The 102-probe sets identified from training data set 1 showed a strong association with patients overall survival (OS). Individual Kaplan-Meier graphs were then evaluated and the significant 92 probe sets were finally considered the prognostic signature (Figure 1) in which 75 probes sets were down-regulated (HR < 1.0) while 17 were up-regulated (HR > 1.0) in patients with breast cancer early deaths (Supplementary Table S3). These 92 probe sets corresponded to 70 annotated gene symbols, 31 were biologically functioning genes, 10 genes were represented by more than one probe set and 8 were unknown genes (Table 6 and Supplementary Table S3). The survival risk prediction analysis was performed to classify patients into two risk groups and generated distinct prognostic index for each patient using all the 92-probe expression values and OS (months) (Figure 2A). The patients were then dichotomized into groups of high or low risk using the 50^th^ percentile (median) cutoff of the prognostic index (−0.272144). To evaluate patient's prognosis, Kaplan-Meier plots were drawn and the log-rank test showed significant differences in all prognostic variables including OS and relapse free survival (RFS) in the training group (*p* < 0.001; Figure 2D and 2E). The heatmap of the 92-probe signature and the clinical variables between risk groups are in Figure 2C and 2B, in which the clusters are correlated with estrogen receptor (ER) and tumor grade but not with nodal status. To evaluate the strength of the predictor, the survival risk prediction analysis was performed separately for the datasets 2 and 3 using both the 102 and the 92-probe sets, respectively. The new prognostic indexes were then generated using survival time [OS and disease free survival (DFS)], and probe expression of individual patients. It is noteworthy that all the results were found to be significantly associated with patients' prognosis (Figure 3).

### Validation of the gene expression signature in independent and combined data sets

**Figure 4:**
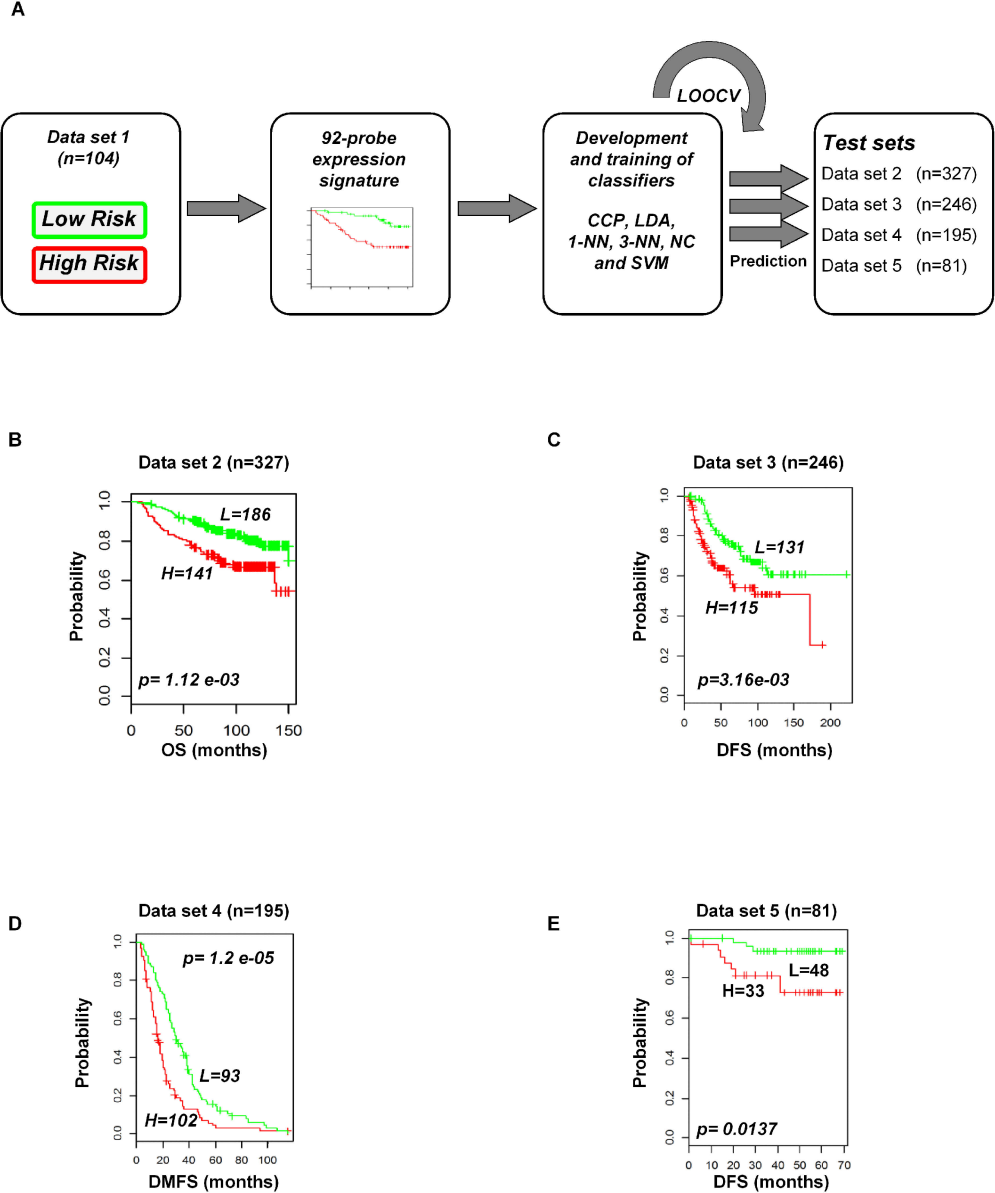
Construction of prediction model in test cohorts based on gene expression signature from data set 1 **A.** Schematic overview of the strategy used for the construction of prediction models and evaluation of predicted outcomes depending on the 92-probe signature. **B–E.** Kaplan-Meier plots of survival graph. According to survival time, patients were stratified into two risk-subgroups, predicted by CCP. (A) Overview of the prognostic signature validation strategy. (B) Dataset 2. (C) Dataset 3. (D) Dataset 4. (E) Dataset 5. *p* values were obtained from log-rank test. The ‘+’ symbols in the panels indicate censored data. CCP, compound covariate predictor; OS, overall survival; DFS, disease free survival; DMFS, disease metastasis free survival.

**Figure 5:**
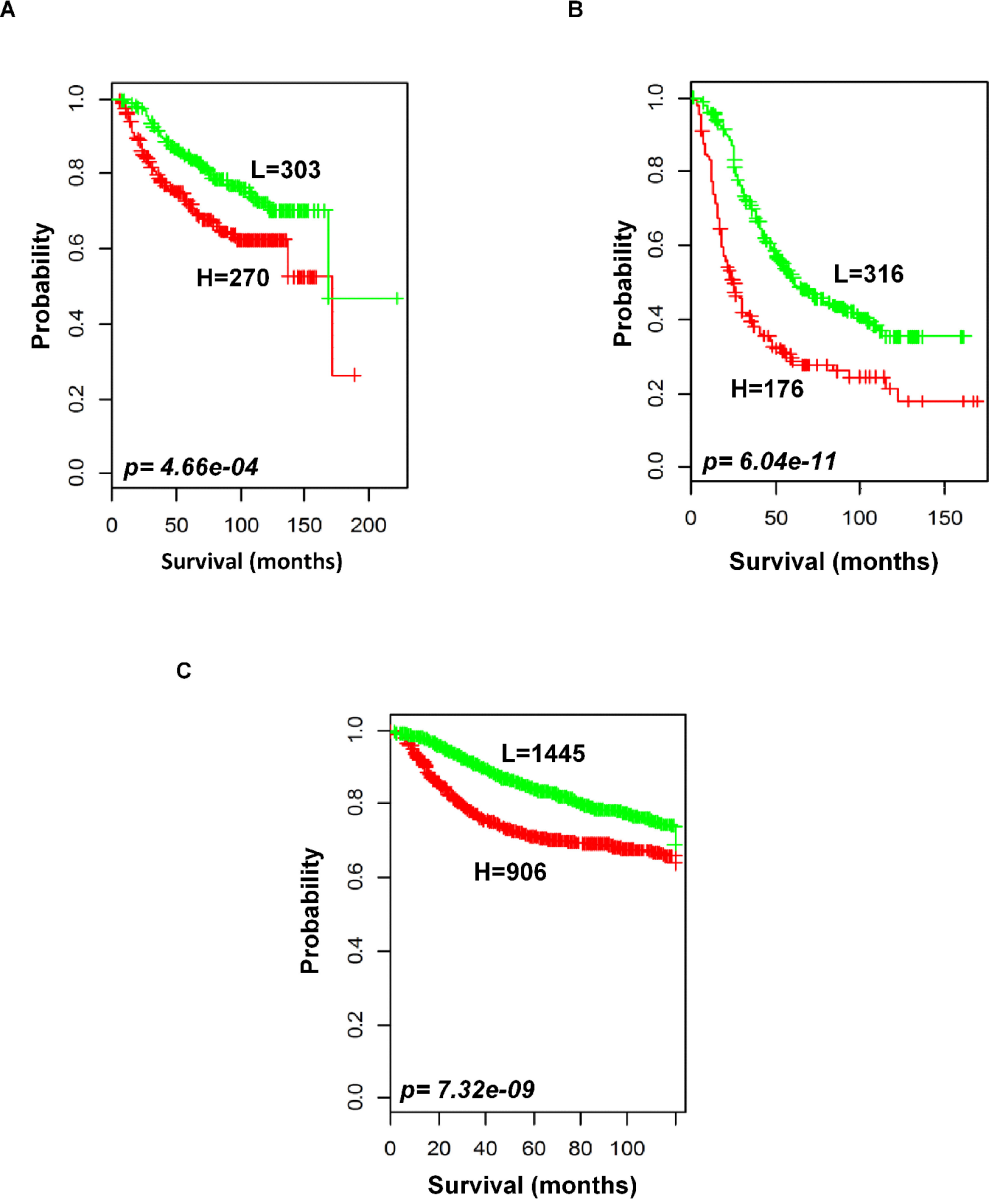
Outcome predictions in the combined validation cohorts Kaplan-Meier survival curves were constructed using 92-probe expression from the training dataset. **A.** Combination of data sets 2 and 3. **B**. Five other plus 2 chip combination. **C.** Ten affymetrix U133A chip combination. Patients were stratified according to median prognostic index into two risk subgroups predicted by CCP. *p* values were obtained from log-rank test. The ‘+’ symbols in the panels indicate censored data.

To evaluate the prognostic performance of the newly developed 92-probe signature, validations were first done on independent datasets 2, 3, 4 and 5. Data set 1 from Ireland cohorts was used for training of classifiers to validate all the datasets. To begin with, the 92 probes of the training cohorts were combined with corresponding probes from each of the validation sets. All the genes in the signature were submitted into the prediction algorithms CCP, LDA, 1NN, 3NN, NC and SVM for the validation of the datasets. Performance of the gene signature was assessed by leave-one-out cross-validation (LOOCV) to obtain the accuracy, sensitivity and specificity. During LOOCV, the specificity for predicting high risk in dataset 2, 3 and 5 was 1.0, while that for dataset 4 was 0.93. On the other hand, the sensitivity of the corresponding datasets was 0.902, 0.961, 0.922 and 0.872, respectively. The area under curve (AUC) during cross validation was 0.999 for the data set 2, 3, and 5; while 0.967 for data set 4 (data not shown). The Kaplan-Meier plots predicted by CCP showed significant difference with prognosis in all independent datasets (*p = 1.12 × 10^−3^*, *p = 3.16 × 10^−3^*, *p = 1.2 × 10^−5^* and *p = 1.37 × 10^−2^*, respectively; Figure 4B–4E). With the exception of SVM of data set 5, all prediction algorithms used in the analysis showed similar strength in significance level (Supplementary Figure S3). To determine whether the signature would improve the prognostic prediction with increased patient population, three subgroups were made for combined validation (see Methods) from 17 breast cancer data sets using Affymetrix U133 plus 2.0 and U133A platforms (Table 1 and Supplementary Table 1). For this purpose, predictions of the signature were done for each subgroup in isolation and for the U133A chip that included only 50 probes to construct the prediction models. As expected, all the prediction methods showed highly predictive performance with more than 95 percent predictive accuracy for all the classifiers (data not shown) and the Kaplan-Meier revealed significant differences of each of the combined data sets (*p = 4.66 × 10^−4^*, *p = 6.04 × 10^−11^* and *p = 7.32 × 10^−9^*, respectively; Figure 5). The signature distinguished 270 (47.1%), 170 (36%) and 906 (38.5%) as the high risk and 303 (52.9%), 316 (64%) and 1, 445 (61.5%) as the low risk for patients' survival rate in subgroups 1, 2 and 3, respectively (Figure 5). Taken together, these data indicate that the selected gene signature might well reflect the patients' potential for survival.

### The 92-probe signature is an independent risk factor for survival

The prognostic accuracy was estimated by the univariate- and the multivariate-Cox proportional hazards models. In Univariate analysis, the 92-probe signature was shown to be a significant indicator for survival [(HR) 1.927 (1.237–3.002); *p* < 0.01]. Of these clinical variables, the tumor grade and p53 status were also associated with better prognosis (*p* < 0.01 and *p* < 0.05 respectively; Table 3.) However, in the multivariate analysis, the molecular subtype and the 92-probe signature were found to be associated significantly with survival [(HR) 1.799 (1.272–2.544); 7.125 (2.462–20.618), *p* = 001; *p* < 0.001]. To evaluate the independent prognostic performance with increased patient number, only those data sets with sufficient clinical and survival information were combined. In this condition, two multivariate Cox models were constructed entering the independent variables of age, ER-, progesterone receptor (PR)-status, lymph node, grade and the absence or presence of the 92-probe signature. In the first model, lymph node and grade were significantly correlated with patients' survival (*p* < 0.01) (Table 4). When the 92-probe signature was added into the second model, the signature was found to be significantly associated with survival. The multivariate analysis was then repeated to assess 5-year DFS. The result revealed the 92-probe signature might be the strongest and most significant predictor of the survival in late recurrence [2.239 (1.265–3.963); *p* = 0.006; Table 5]. In order to further confirm this association with early- or late-recurrence of the disease, the patients were grouped according to follow-up time (A: ≤ 5 yr, B: ≥ 5 yr). The signature retained a significant association only in case of patients in the B group, and lymph node status was the significant predictor of survival among those clinical variables (Supplementary Table S2).

**Table 3:** The univariate and the multivariate cox proportional hazard regression analyses for patients' survival in France cohort

Parameters (*n* = 246)	Univariate		Multivariate	
	HR (95%CI)	*p* Value	HR (95%CI)	*p* Value
Age (years)	0.997 (0.980–1.015)	0.756	0.990 (0.969–1.010)	0.328
ER status (+/–)	0.687 (0.440–1.072)	0.098	0.977 (0.337–2.833)	0.965
PR status (+/–)	0.816 (0.524–1.270)	0.368	1.349 (0.527–3.455)	0.533
Lymph node (+/–)	1.493 (0.952–2.341)	0.081	1.357 (0.794–2.317)	0.264
Grade (1, 2, 3)	1.592 (1.159–2.188)	0.004	0.912 (0.605–1.376)	0.661
P53 status (yes/no)	1.814 (1.100–2.991)	0.020	1.378 (0.792–2.399)	0.257
Mol_Sub (I, II, III, IV, V, VI)	1.037 (0.875–1.229)	0.677	1.799 (1.272–2.544)	0.001
92-probe signature (high/low)	1.927 (1.237–3.002)	0.004	7.125 (2.462–20.618)	<0.001

HR, hazard ratio; CI, confident interval; ER, estrogen receptor; PR, progesterone receptor; Mol_Sub, molecular subtype; A low risk was defined as a prognostic index less than or equal to −0.272144, and a high risk as a PI higher than −0.272144.

**Table 4:** Multivariate analysis of age, ER-, PR-status, lymph node, grade and 92-probe signature in relation to the patient's survival

Parameters	HR (95%CI)	*p* Value
**Analysis without 92-probe signature**		
**Age (years)**	0.996 (0.980–1.013)	0.674
**ER status (+/–)**	0.570 (0.269–1.204)	0.141
**PR status (+/–)**	1.148 (0.555–2.371)	0.710
**Lymph node (+/–)**	1.799 (1.161–2.788)	0.009
**Grade (1, 2, 3)**	1.536 (1.121–2.105)	0.008
**Analysis with 92-probe signature**		
**Age (years)**	0.996 (0.979–1.014)	0.674
**ER status (+/–)**	1.111 (0.479–2.577)	0.806
**PR status (+/–)**	1.269 (0.610–2.639)	0.524
**Lymph node (+/–)**	1.856 (1.197–2.876)	0.006
**Grade (1, 2, 3)**	1.382 (1.000–1.908)	0.050
**92-probe signature (low/high)**	2.746 (1.443–5.227)	0.002

The multivariate model included 301 patients for DFS, owing to missing values in twenty two. A low risk was defined as a prognostic index less than or equal to −0.272144, and a high risk as a PI higher than −0.272144. HR, hazard ratio; CI, confident interval; ER, estrogen receptor; PR, progesterone receptor.

**Table 5:** Multivariate analysis of age, ER-, PR-status, lymph node, grade and 92-probe signature in relation to the 5-year survival

Parameters	HR (95%CI)	*p* Value
**Analysis without 92-probe signature**		
**Age (years)**	0.984 (0.971–0.998)	0.025
**ER status (+/–)**	0.510 (0.270–0.961)	0.037
**PR status (+/–)**	0.905 (0.479–1.710)	0.757
**Lymph node (+/–)**	1.517 (1.062–2.165)	0.022
**Grade (1, 2, 3)**	1.708 (1.291–2.258)	<0.001
**Analysis with 92-probe signature**		
**Age (years)**	0.984 (0.970–0.998)	0.023
**ER status (+/–)**	0.878 (0.423–1.825)	0.728
**PR status (+/–)**	0.974 (0.512–1.853)	0.936
**Lymph node (+/–)**	1.539 (1.079–2.197)	0.017
**Grade (1, 2, 3)**	1.562 (1.173–2.080)	0.002
**92-probe signature (low/high)**	2.239 (1.265–3.963)	0.006

A low risk was defined as a prognostic index less than or equal to −0.272144, and a high risk as a PI higher than −0.272144. HR, hazard ratio; CI, confident interval; ER, estrogen receptor; PR, progesterone receptor.

### Significant association of prognosis in estrogen receptor positive and lymph node positive patients

**Figure 6:**
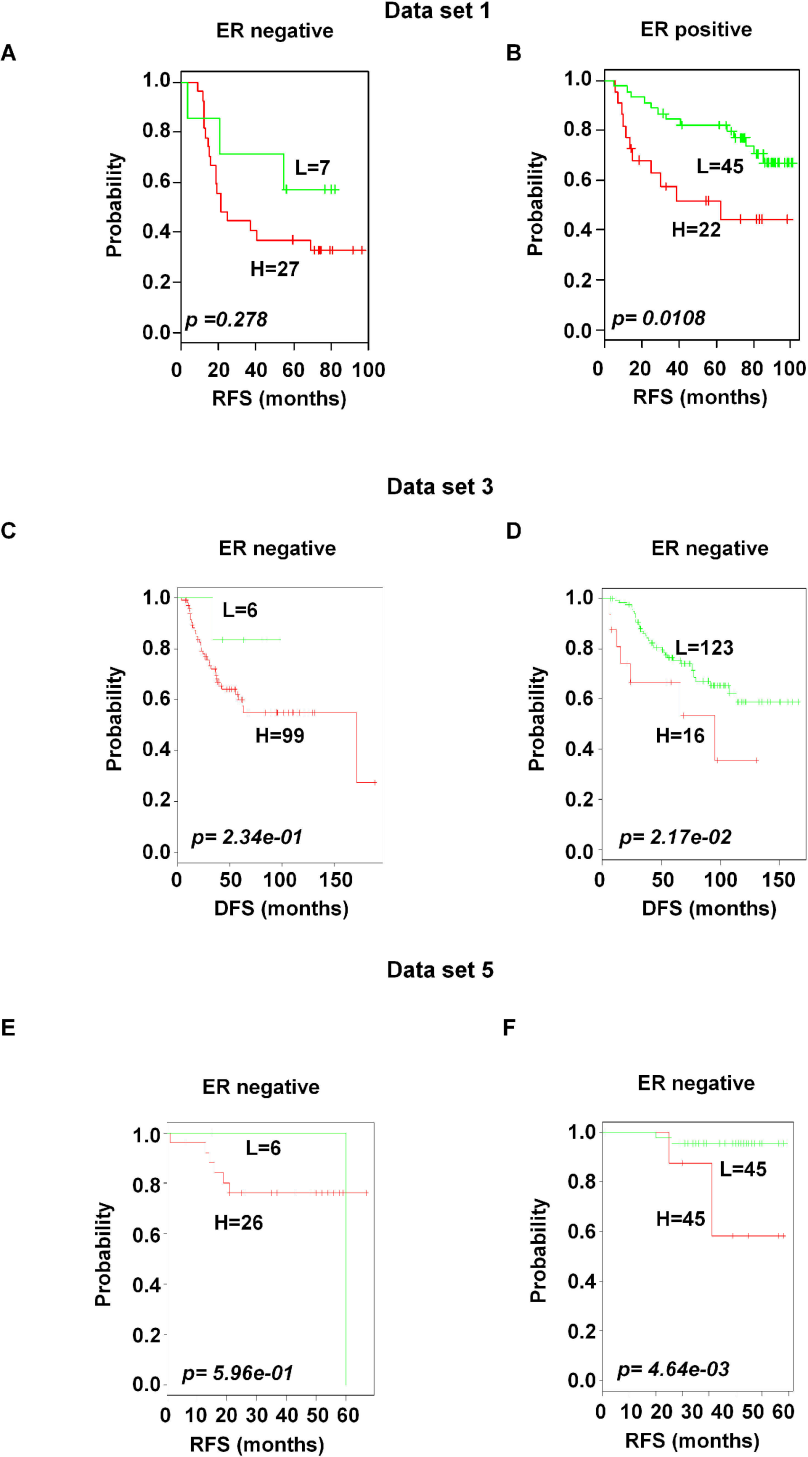
Significant association of the 92-probe signature with ER status in different datasets **A–F.** Kaplan-Meier curves of patients in ER-negative and ER-positive groups. Patients were classified according to the prognostic index of the 92-probe signature. (A and B) Dataset 1. (C and D) Dataset 3. (E and F) Dataset 5. *p* values were obtained from log-rank test. The ‘+’ symbols in the panels indicate censored data. DFS, disease free survival; RFS, relapse free survival.

**Figure 7:**
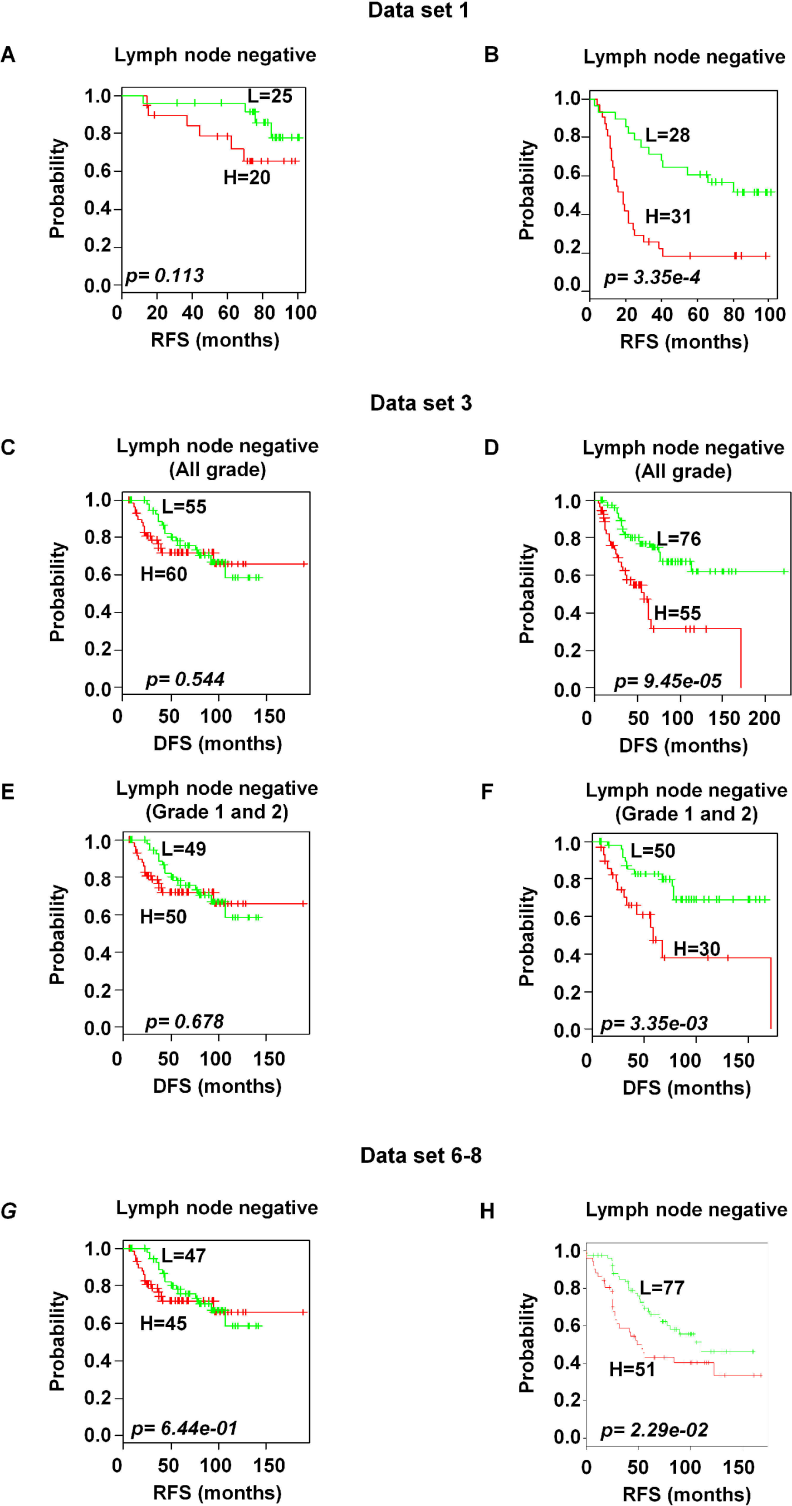
Significant association of 92-probe signature with lymph node status in different datasets **A–H.** Kaplan-Meier curves of patients in lymph node-negative and lymph node-positive groups. Patients were classified according to the prognostic index of the 92-probe signature. (A and B) Dataset 1. (C and D) Dataset 3 including all tumor grades. (E and F) Dataset 3 including tumor grade 1 and 2 or pT1 and pT2. (G and H) Canada cohorts including datasets (6, 7 and 8). *p* values were obtained from log-rank test. The ‘+’ symbols in the panels indicate censored data. DFS, disease free survival; RFS, relapse free survival.

Based on the available clinical information and sufficient patients' number, subset analyses were performed within the ER and lymph node status. The gene expression signature successfully identified patients with poor survival among those with ER-positive and positive axillary lymph node involvement in breast cancer within the training data set 1 (*p* < 0.05 and < 0.001 for ER-and lymph node positive respectively; Figure 6B and 7B). A distinctive subgroup within the ER-positive was found to be significantly associated with DFS in the independent datasets (*p* < 0.05, *p* < 0.01; data sets 3 and 5 respectively; Figure 6). The lymph node analysis showed strongly association with survival both in independent and combined datasets (*p* < 0.001, dataset 3 and combined cohorts AL; *p* < 0.05, Canada cohorts; Figure 7 and Supplementary Figure 4). When we considered tumor grade 1 and 2 or stage pT1 and pT2, the significant association with lymph node was well maintained. Taken together, these results suggest that the gene expression signature is independent of the current ER and lymph node status.

### Biological significance and gene interaction

**Figure 8:**
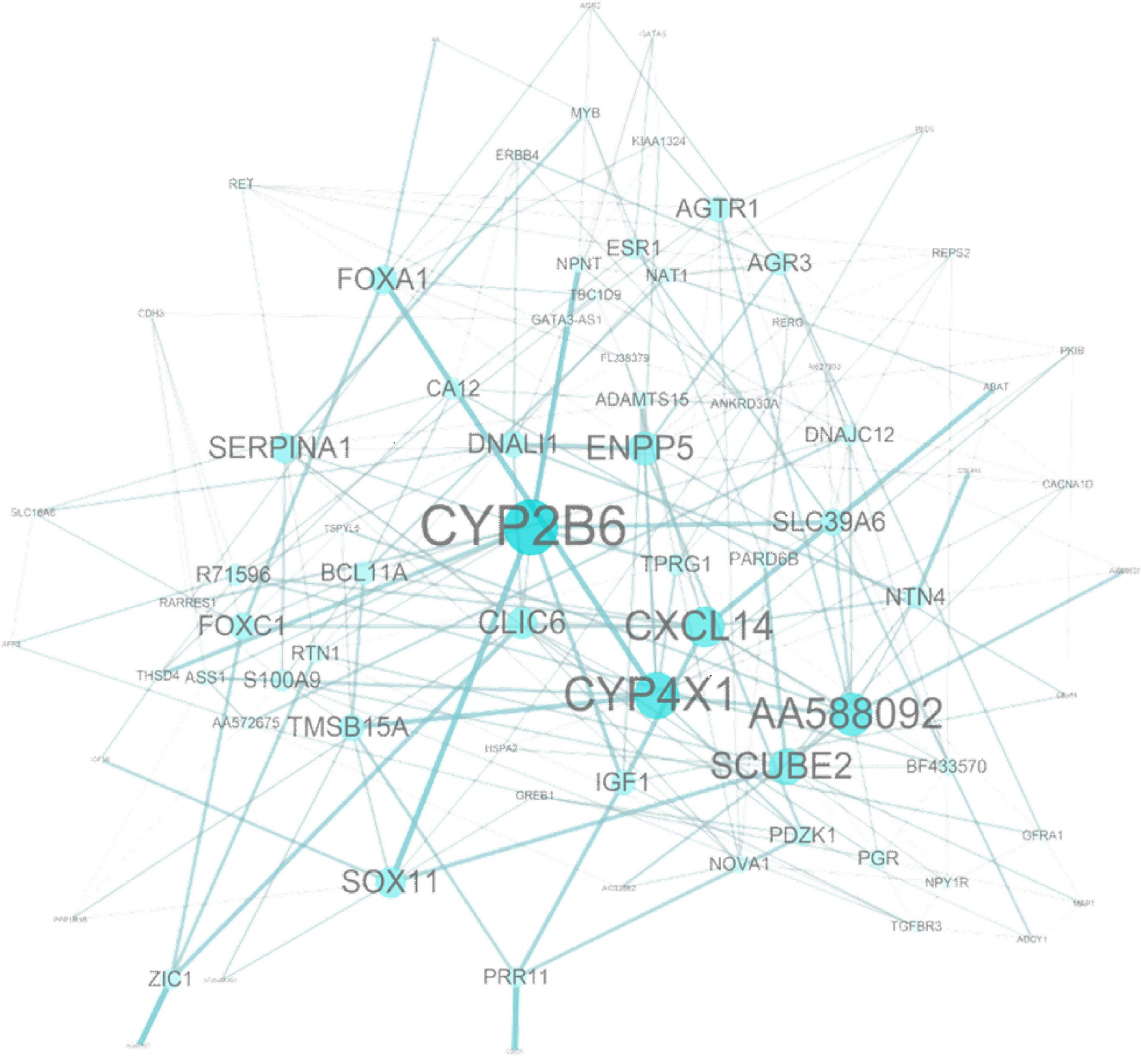
Network analysis of the 92-probe signature in the primary breast cancer Node and edge size were generated according to the number of connections within the module.

The function of the 92 probes in the prognostic signature was analyzed to relate the genes to biological processes. Eight probes were not annotated genes. The enriched biological processes were shown in Table 6. The most significant biological process was a response to hormone stimuli (*p* < 0.001) in which 9 genes were adenylate cyclase 1 *(ADCY1),* erb-b2 receptor tyrosine kinase 4 *(ERBB4)*, estrogen receptor 1 *(ESR1),* GATA binding protein 3 *(GATA3),* insulin-like growth factor 1 receptor *(IGF1R),* neuropeptide Y receptor Y1 *(NPY1R)*, ras-related and estrogen-regulated growth inhibitor-like protein *(RERG),* serpin peptidase inhibitor clade A member 1 *(SERPINA1)*, and transforming growth factor beta receptor III *(TGFBR3)*. Two additional hormonal processes, namely responses to steroid hormone stimulus and estrogen stimulus, were found. While cell motion was the least significant process (*p* < 0.05), it consisted of 6 genes such as axonemal dynein light intermediate polypeptide 1 *(DNALI1)*, forkhead box C1 (*FOXC1),* insulin-like growth factor 1 *(IGF1),* ret proto-oncogene *(RET),* S100 calcium binding protein A9 *(S100A9), and TGFBR3*. Other important processes like gland development, response to organic substance, regulation of epithelial cell proliferation, cell maturation, mesenchymal cell differentiation, regulation of cell migration along with some signaling pathways such as protein tyrosine kinase signaling and second-messenger-mediated signaling pathways were found to be considerably predominating biological processes. Genes involved in multiple biological processes were *FOXC1, TGFBR3, IGF1R, IGF1, RET, NPY1R* and forkhead box A1 *(FOXA1)*, which participated in 7 to 15 processes while protein phosphatase 1 regulatory subunit 1B (*PPP1R1B*) and zinc finger protein ZIC 1 *(ZIC1)* included only single cell behavior process. A network analysis of the 92-probes showed cytochrome P450 2B6 *(CYP2B6)* is linked to the strongest protein-protein interaction especially with cytochrome P450 4X1 *(CYP4X1), FOXA1, IGF1,* nephronectin *(NPNT),* and transcription factor SOX-11 *(SOX11)*. In addition, an unknown gene *(AA588092),* anterior gradient homolog 3 *(AGR3),* angiotensin II receptor type 1 *(AGTR1)*, chloride intracellular channel protein 6 *(CLIC6),* C-X-C motif chemokine 14 *(CXCL14),* ectonucleotide pyrophosphatase/phospho-diesterase 5 *(ENPP5), FOXC1,* signal peptide-CUB-EGF-like domain containing protein 2 *(SCUBE2),* and *SERPINA1* were also found to be strongly connected within the signature (Figure 8).

**Table 6:** The significant GO biological pathways pointed to by the 92-probe signature

ID	Name	No of genes	*p* value	Gene symbol
GO:0009725	Response to hormone stimulus	9	0.000124	ADCY1, IGF1R, GATA3, RERG, TGFBR3, SERPINA1, ERBB4, NPY1R, ESR1
GO:0048732	Gland development	6	0.000233	IGF1, IGF1R, PGR, FOXC1, ERBB4, FOXA1
GO:0010033	Response to organic substance	11	0.000665	HSPA2, ADCY1, GATA3, IGF1R, ABAT, RERG, SERPINA1, TGFBR3, ERBB4, NPY1R, ESR1
GO:0021700	Developmental maturation	5	0.000798	PGR, ERBB4, NTN4, RET, FOXA1
GO:0001655	Urogenital system development	5	0.001098	FOXC1, AGTR1, SOX11, RET, FOXA1
GO:0030879	Mammary gland development	4	0.002238	IGF1, IGF1R, PGR, ERBB4
GO:0007610	Behavior	8	0.003072	ADCY1, ABAT, PPP1R1B, ZIC1, CXCL14, NOVA1, NPY1R, S100A9
GO:0050678	Regulation of epithelial cell proliferation	4	0.003147	IGF1, PGR, TGFBR3, ERBB4
GO:0048469	Cell maturation	4	0.003675	PGR, NTN4, RET, FOXA1
GO:0030334	Regulation of cell migration	5	0.00521	IGF1, IGF1R, TGFBR3, ERBB4, PARD6B
GO:0001822	Kidney development	4	0.007315	FOXC1, AGTR1, SOX11, RET
GO:0048545	Response to steroid hormone stimulus	5	0.008129	GATA3, SERPINA1, ERBB4, NPY1R, ESR1
GO:0051270	Regulation of cell motion	5	0.008276	IGF1, IGF1R, TGFBR3, ERBB4, PARD6B
GO:0043627	Response to estrogen stimulus	4	0.00935	GATA3, SERPINA1, NPY1R, ESR1
GO:0040008	Regulation of growth	6	0.013202	IGF1, RERG, FOXC1, AGTR1, NPY1R, MAPT
GO:0007167	Enzyme linked receptor protein signaling pathway	6	0.013356	IGF1R, REPS2, FOXC1, TGFBR3, ERBB4, RET
GO:0007169	Transmembrane receptor protein tyrosine kinase signaling pathway	5	0.013730	IGF1R, REPS2, FOXC1, ERBB4, RET
GO:0019932	Second-messenger-mediated signaling	5	0.016108	ADCY1, IGF1, IGF1R, AGTR1, NPY1R
GO:0014031	Mesenchymal cell development	3	0.018849	FOXC1, TGFBR3, RET
GO:0048762	Mesenchymal cell differentiation	3	0.018849	FOXC1, TGFBR3, RET
GO:0060485	Mesenchyme development	3	0.019552	FOXC1, TGFBR3, RET
GO:0002070	Epithelial cell maturation	2	0.020530	PGR, FOXA1
GO:0003006	Reproductive developmental process	5	0.023000	HSPA2, IGF1R, PGR, FOXC1, FOXA1
GO:0007626	Locomotory behavior	5	0.026563	ABAT, CXCL14, NOVA1, NPY1R, S100A9
GO:0014855	Muscle cell proliferation	2	0.028626	FOXC1, TGFBR3
GO:0008283	Cell proliferation	6	0.033837	IGF1, PDZK1, FOXC1, TGFBR3, ERBB4, SOX11
GO:0030182	Neuron differentiation	6	0.034411	IGF1R, RTN1, NTN4, RET, PARD6B, FOXA1
GO:0033002	Muscle cell proliferation	2	0.036656	FOXC1, TGFBR3
GO:0008015	Blood circulation	4	0.041680	ABAT, FOXC1, AGTR1, NPY1R
GO:0042127	Regulation of cell proliferation	8	0.042679	IGF1, IGF1R, RERG, PGR, AGTR1, TGFBR3, ERBB4, RARRES1
GO:0006928	Cell motion	6	0.046139	IGF1, FOXC1, TGFBR3, RET, DNALI1, S100A9

*p*-value represents the significance of enrichment and is estimated by Bonferroni test.

## DISCUSSION

In this study, we explored a significant gene signature related to prognosis of breast cancer patients by investigating three independent microarray datasets of heterogeneous primary breast cancer. The reproducibility of the signature was improved by using a unique platform and probe ID, and repeated analysis strategies were followed. By selecting common genes before the Univariate Cox, we controlled the gene set instability and overfitting of the training [13]. In addition, we improved the predictive strength by analyzing 677 patients without pooling microarray data sets. A supervised method was applied to construct the signature, test its robustness and validate its association with clinical outcomes. The two risk subgroups based on the prognostic index significantly reduced overoptimization with 73% overall accuracy during cross validation of the training dataset. Subsequent analysis of the clinical data revealed that the two subgroups differed significantly in OS and RFS. Although our strategy might lose some predictive power by analyzing 3 data sets separately (Figure 3), the predictive strength was fairly conserved by showing significant association with patients' prognosis in dataset 2 and 3, using both 102 and 92 probe sets.

The 92-probe signature was robustly validated by six different predictors in internal, external and combined approaches with large numbers of patients, and almost all showed a similar performance. The robust validation was supported by the high sensitivity (>90%) and specificity (>90%) of all the prediction models within dataset 1 and a significant association of predicted outcomes was found with patient prognosis in all independent datasets (2, 3, 4, and 5) (Figure 4, 4B–4E and Supplementary Figure S3, S3A–S3D). Other datasets of GPL 570 chip were not considered for independent validation because of small sample size. Datasets 2 and 3 were considered for independent validation because the survival information remained intact during identification of the common genes. Due to this reason, we made a separate subgroup by pooling only the datasets 2 and 3 for combined validation. Interestingly, other two subgroups showed better prediction for survival (Figure 5). The combined validation approach showed a better predictive performance suggesting that the number of patient population is equally important to confirm the validity of a signature. Although half of the probe sets were able to validate the outcome prediction when the sample size was more than 2000 (Figure 5C), the present study mainly focused on full probe models. We followed a strict protocol to maintain the same analytical method in training and validation. Our prognostic index based gene signature worked well in diverse populations of primary breast cancers, suggesting that it has an important general prognostic feature.

Different multivariate analyses were performed in the present study to explore whether the prognostic value was conserved after adjusting the clinical variables. In univariate Cox analysis, our 92-probe signature showed independent predictive power of patients' survival, while the predictive performance increased after adjustment of clinical variables in multivariate models. Analysis based on two multivariate models containing clinical variables, with and without the signature, (Table 4) shows a significant association of the 92-probes with survival, indicating the true predictive power [14, 15]. Finally, we tested whether it might predict early- or late-recurrences of the disease. The association of the signature with 5-year disease free survival indicated that the signature was a predictor of late recurrence, and further demonstrated that only a sub-group of patients were at increased risk for this recurrence (Supplementary Table S2). It is notable that in multivariate analysis, the predictive power of molecular subtype increased significantly in single data while lymph node and grade were significant predictors in increased patient data, suggesting an integrated approach using gene expression together with clinical information might be more promising in clinical practice.

A functional enrichment analysis showed that the 92-probe signature was significantly enriched in hormone response, mammary gland development, response to steroid hormone and the estrogen stimulus response. These important hormonal regulators permitted us to analyze the gene signature's effects on the ER status. The two risk groups in ER-positive patients indicated that distinct biological characteristics were reflected by gene expression patterns representing heterogeneity of aggressiveness. For example, low level of MAPT expression found in the present study was associated with a subset of ER-positive breast cancers that had poor prognosis with tamoxifen and might benefit from taxane-containing chemotherapy [16]. One important finding of our study is that the proportion of high-risk patients in the training and validation sets was lower than that of the low risk patients. The large number of patients (50-probe sets) also showed consistent results (Figure 6 and Supplementary Figure S4) suggesting that minorities of ER+-primary breast cancer patients have likelihood for poor prognosis. This needs to be considered for therapeutic decisions to protect patients from overtreatment.

Metastatic relapse mostly depends on large tumor size, high-grade and positive lymph node status [17]. Notable findings in our study are that (a) the signature divided lymph node positive breast cancer into two risk groups, and (b) the signature was robustly validated in different patients' cohorts (Figure 7). This prognostic index remained significant even when we excluded higher tumor grade patients (Figure 7F), suggesting that the signature has the potential to predict invasiveness from early stage in this group. The invasive feature has also been shown in the data sets 2; about 70% patients received adjuvant chemotherapy before samples were taken, indicating genes in the signature are involved in either resistance or low effectiveness to chemotherapy. The signature included cell proliferation-related genes such as *RERG, CDC20* and voltage-dependent L-type calcium channel subunit alpha-1D *(CACNA1D)*. This is consistent with elevated cell proliferation and loss of cell cycle control associated with poor outcomes [18–21]. Cell migration plays an important role in metastasis from epithelial to mesenchymal transition. The overexpression of P-cadherin induces cell migration and promotes cell invasion by disrupting the interaction between E-cadherin and cytoplasmic catenins [22]. In addition, prognostic biomarkers *FOXC1* and *TGFBR3* showed significant association with poor survival. This is in line with Ray *et al* [23] and Dong *et al* [24] who demonstrated that these genes function in relation to breast cancer cell growth, migration, invasion, and chemoresistance. Therefore, we argue that the varieties of genes in this signature are involved in infinitive proliferation, metastasis and chemoresistance. Many new genes such as *AA588092, AI367357*, uncharacterized FLJ38379 (FLJ38379), tumor protein p63 regulated 1 *(TPRG1)* and a disintegrin and metalloproteinase with thrombospondin motifs 15 *(ADAMTS15)* were also found, suggesting that our 92-probe signature contains novel information which may provide new biomarkers to assist in clinical decision making regarding new therapeutic targets for the disease.

The signature lacks *BRCA1, BRCA2, p53, Ki67*, and some other important genes that are causally related to breast cancer development. One possible explanation could be that data sets were mainly generated from luminal type breast cancer. Another cause may be due to the confined folds and observations. Inter-laboratory variations can also skip some genes during filtration steps. Even if this approach might have some limitations, we found several molecules of key signaling pathways in cancer metabolism. For example, the downregulation of CXCL14 upregulated CXCL12, which in turn activated DARPP-32 that mediated invasion via CXCR4 [25, 26]. A few genes such as the survival mediator *RET* and *N-acetyltransferase* showed opposite findings, suggesting the importance of reinvestigating pathophysiology of early- and late-recurrences before selecting the new therapeutic target. Unfortunately, our study demonstrated a lack of many overlapping genes between our gene signature and existing gene signatures (Supplementary Table S4) [5, 27–30]. But this discrepancy is a very common phenomenon in Microarray analyses. This could be due to small sample size, patients' characteristics, statistical analyses, different platforms with different methodologies for tumor collection and RNA preparation, and relative quantification values for a given gene. All these factors might explain the lack of common genes among published signatures. In our study we put most of these factors into consideration though further work needs to be done to come up with more overlapping genes for better diagnosis and treatment.

In conclusion, we suggest that a prognostic 92-probe signature is developed to predict outcome in primary breast cancer. This signature may stratify subgroups of breast cancer patients with poor prognosis in a reliable and reproducible manner across independent and combined patients' cohorts. Our data suggests that this classifier may have a considerable clinical relevance, especially in identifying patients at high risk of developing late recurrences. This gene profiling can preferentially be valuable as a target for prognosis and treatment of ER-positive and lymph node positive patients. This study provides an opportunity for a rational design of future clinical trial to test the benefit against late recurrences in these groups of patients.

## METHODS

### Patients and gene expression data

In the current analysis, 18 different breast cancer patient datasets were studied. Gene expression datasets were downloaded from the GEO repository (http://www.ncbi.nlm. http://nih.gov/geo), Array express (https://www.ebi.ac.uk/arrayexpress/experiments) and The Cancer Genome Altas (TCGA) (http://cancergenome.nih.gov/). Data were selected based on the chip type [Affymetrix U133 2.0 (GPL570) and HG-133A (GPL96)], raw CEL files and clinical survival information. Multiple data from the same institution were excluded, except for three datasets, GSE9195, GSE20711 and GSE16391, from Princess Margaret Research, Canada. The raw CEL files were preprocessed with robust multiarray average (RMA) algorithm [31] using R packages from ‘affy’ Bioconductor (http://www.bioconductor.org). Based on the U133 plus 2.0 platform and patients characteristics, five datasets were mainly considered in the study. To identify the survival gene candidates, dataset 1 (GSE42568) from Dublin City University (Ireland) was used as a training cohort consisted of 121 patients, 17 of whom were excluded due to normal breast tissue. Datasets 1 to 3 were used for the identification of common genes while datasets 2 to 5 were used as independent testing samples for validation (internal and external) of the identified signature. The first validation dataset 2 (GSE20685, *n* = 327) was published by the Koo Foundation SYS Cancer Center, Taiwan. Dataset 3 (GSE31448, *n* = 357) was taken from the Institut Paoli-Calmettes, France, however, only 246 patients that provided detailed survival and clinical information were analyzed in this study. Dataset 4 (GSE12276) was collected from the Netherlands Erasmus Medical Centre and 195 patients out of 204 were analyzed in the present study. Finally, dataset 5 (GSE48390, *n* = 81) was used as the last validation set from the Cathay General Hospital SiJhih, Taiwan. Details about the patients' characteristics were described in Table 2. Other datasets used for the combined validation was described in Supplementary Table S1.

### Development of a prognostic survival signature and risk prediction

The following three steps were included in the identification of the prognostic signature: (1) selection of the common probes using the Venn diagram method (2) determination of the optimal survival gene by the univariate Cox and (3) prognostic prediction between gene expression and patient's survival by supervised principle component method. At first, the 54675 probe sets from data sets 1 to 3 were filtered by the Gene filtration method and the common probes were identified by a Venn diagram generator (http://www.pangloss.com/seidel/Protocols./venn.cgi). The univariate Cox analysis, based on hazards model and Wald statistics [32], was used to identify OS-associated genes from the common probes of the training data set. Finally, the survival signature was selected by significant individual Kaplan-Maier graphs provided by the analysis. As for prognostic prediction, probes from the survival signature were applied to the survival risk prediction analysis [33]. This method used the principal component from the training dataset and produced prognostic index (PI) for each patient. We computed using the formula ∑iWiXi+0.100356, where Wi and Xi were the weight and logged gene expression for the i-th gene. When the prognostic index was larger than the median value (−0.272144), the sample was predicted as one with high risk, while the prognostic index was smaller than or equal to the median value, the sample was predicted as one with low risk.

### Validation of the prognostic signature

The validation of the survival gene signature was accomplished on independent and combined datasets. For the combined validation, three subgroups were made depending on the chip versions and the internal data sets. These included 573 breast cancer patients from the internal test sets, 492 from external test sets and 2351 from the affymatrix U133A version (Figure 5 and Supplementary Figure S5). For lymph node subtypes validation, a cohort (Canada) from datasets 6, 7 and 8 were done (lymph node; negativ*e* = 92, positiv*e* = 128). Under this condition, gene expression data from different cohorts were adjusted individually by subtracting the median expression value across the samples before combining them. Six different prediction methods were applied for the validation of all datasets which included compound covariate predictor (CCP), linear discriminant analysis (LDA), support vector machine (SVM), nearest neighbor 1NN, 3NN and nearest centroid (NC) [34]. The robustness of the classifier was estimated by the misclassification rate determined during the leave-one-out cross-validation (LOOCV) in the training set. The Kaplan–Meier survival analyses were performed after the samples were classified into two risk groups and log-rank tests were used to evaluate the survival risk between two predicted subgroups of patients. The uni- and multi-variate Cox proportional hazard regression analyses were used to evaluate independent prognostic factors associated with survival. And gene signature, tumor grade and pathological characteristics were used as covariates.

### Pathway analysis

Gene ontology (GO) biological process enrichment analysis was carried out using the database for Annotation, Visualization and Integrated Discovery (DAVID) bioinformatics tool (http://david.abcc.ncifcrf.gov/) [35]. A connection index based network of the prognostic gene signature was also generated by R program. Cytoscape was used to visualize the connection of each gene in the survival signature [36].

### Statistical methods of microarray data

Microarray data were analyzed using BRB-Array Tools Version 3.0 (http://linus.nci.nih.gov/BRB-ArrayTools.html) [34]. All other statistical analyses were accomplished in the R language environment (http://www.r-project.org) and Statistical Package for Social Sciences (SPSS) software (version 16, SPSS, Inc, Chicago, IL, USA). All comparisons of Kaplan-Meier survival analysis were performed by the log rank test. Cluster analysis was performed with Cluster and Tree View (http://bonsai.hgc.jp/~mdehoon/software/cluster/software.htm#ctv) [37]. *p* value of less than 0.05 was considered statistically significant.

## SUPPLEMENTARY FIGURES AND TABLES




