# Supplementary material for: Prognostic value of a 92-probe signature in breast cancer
Source: Oncotarget. 2015 Apr 11;6(17):15662–80. doi: 10.18632/oncotarget.3525 (PMC4558178; doi:10.18632/oncotarget.3525)
Supplement: Supplementary file 1 [file oncotarget-06-15662-s001.pdf]

## SUPPLEMENTARY FIGURES AND TABLES

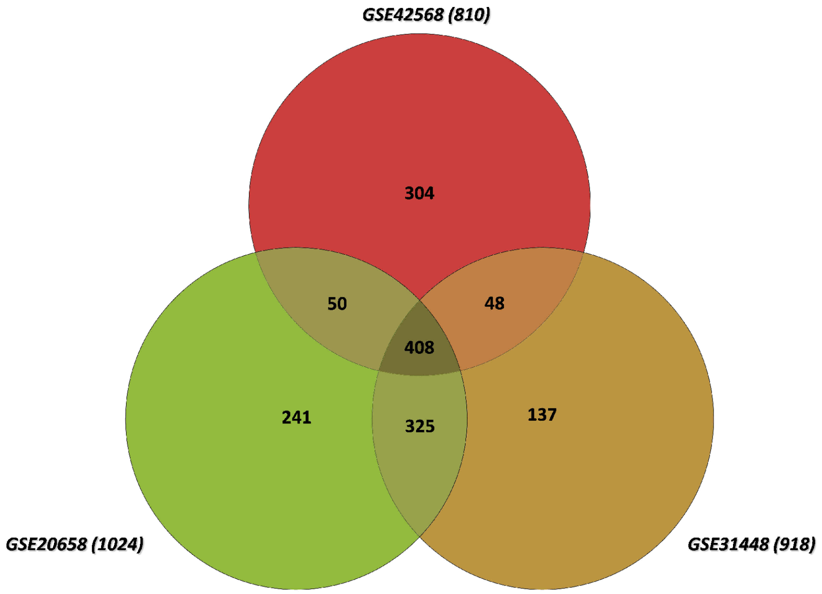


**Supplementary Figure S1: The Common probes among the three different GEO microarray datasets.** The Venn diagram shows overlapping probe sets among data set 1, data set 2, and data set 3, resulting in the 408 common probe sets.

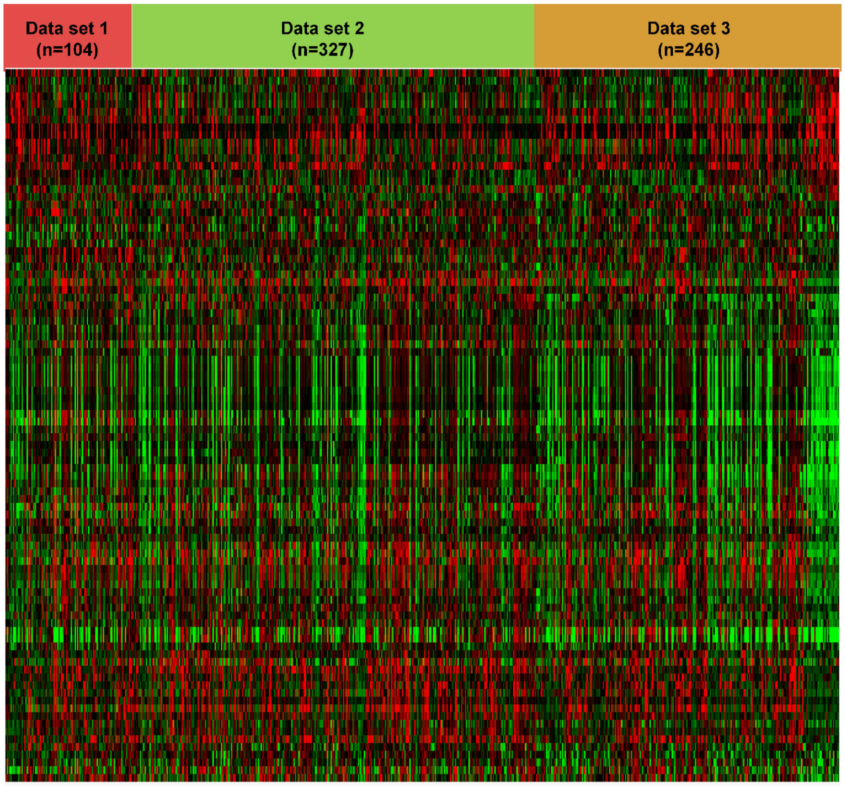


**Supplementary Figure S2: Hierarchical cluster analysis represents the 408 common probe sets expression among the three cohorts.** Each column indicates a patient in the data set, and each row represents a gene in the 408 probes.

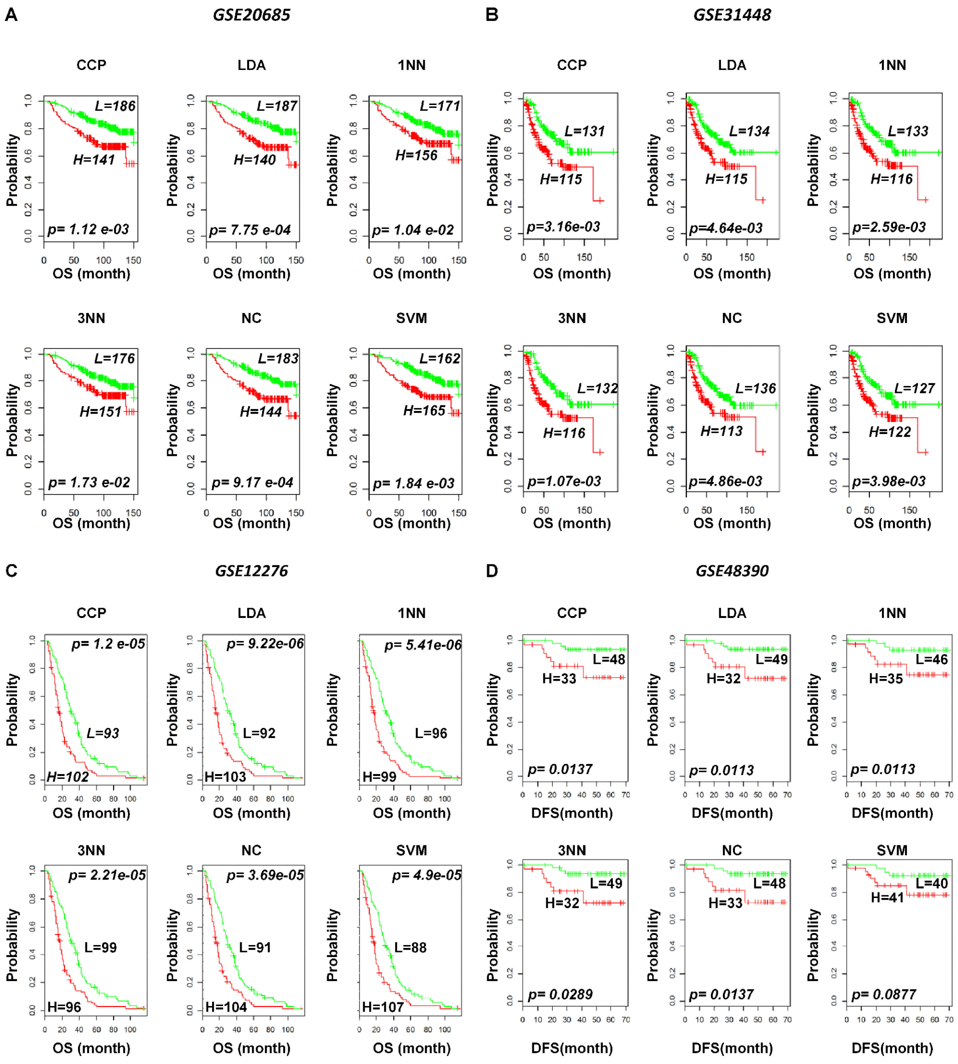


**Supplementary Figure S3: Kaplan-Meier survival curves of four independent validation cohorts.** Datasets 1–4 are shown in figure **A**, **B**, **C** and **D**, and predicted by LDA, 1NN, 3NN, NC and SVM prediction algorithms. *p* values were obtained from log-rank test. The '+' symbols in the panels indicate censored data.

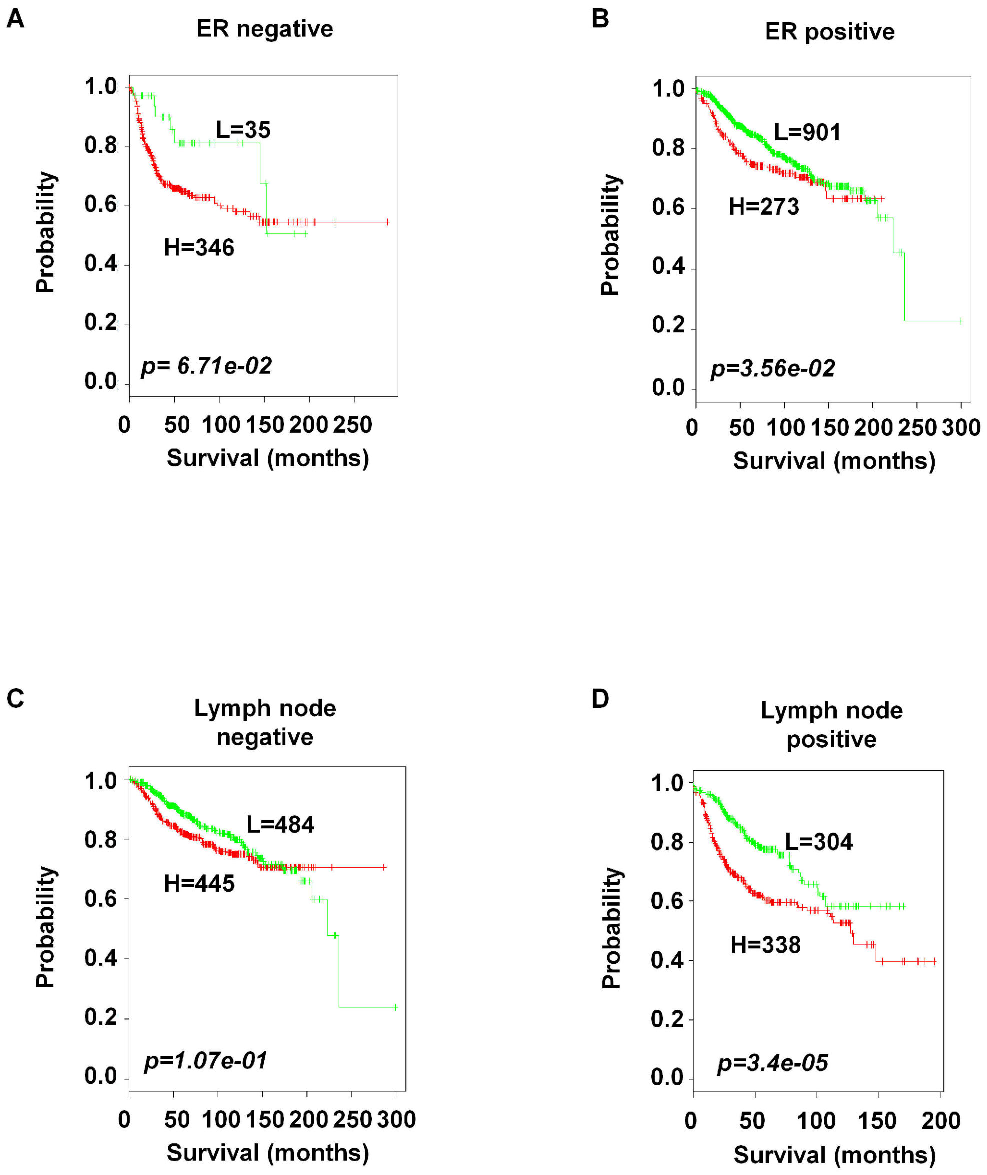


**Supplementary Figure S4: Significant association of 92-probe signature with ER and lymph node in pooled U133A datasets.** **A** and **B.** Kaplan-Meier curves of patients in ER-negative and ER-positive groups. **C** and **D.** Kaplan-Meier curves of patients in lymph node-negative and lymph node-positive groups. Patients were classified according to the prognostic index of the 92-probe signature.

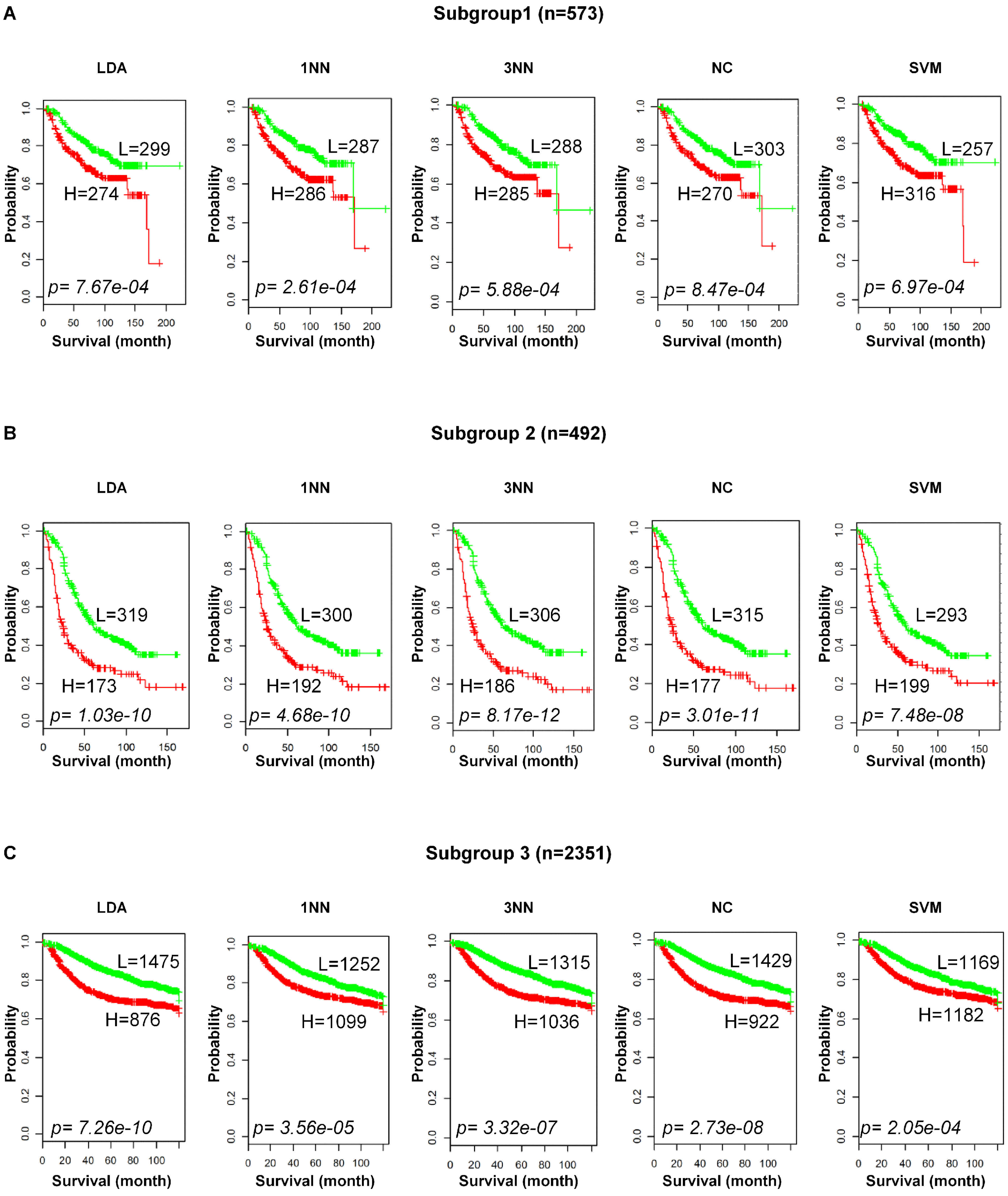


**Supplementary Figure S5: Kaplan-Meier survival curves of three combined validation cohorts predicted by LDA, 1NN, 3NN, NC and SVM prediction algorithms.** **A**. Combination of data sets 2 and 3. **B.** Five external plus 2 chip combination.**C** Ten external U133A chip combination. *p* values were obtained from the log-rank test. The '+' symbols in the panels indicate censored data.

**Supplementary Table S1:** The additional microarray data sets.

| GEO Number | Platform | Author | Paper Title | References |
|---|---|---|---|---|
| GSE20711 | GPL570 | Dedeurwaerder *et al* | Epigenetic portraits of human breast cancers (expression data) | EMBO Mol Med 2011 |
| GSE9195 | GPL570 | Loi *et al* | Predicting prognosis using molecular profiling in estrogen receptor-positive breast cancer treated with tamoxifen | BMC Genomics *2008* |
| GSE16391 | GPL570 | Desmedt *et al* | GGI: a potential predictor of relapse for endocrine-treated breast cancer patients in the BIG 1–98 trial | BMC Med Genomics 2009 |
| GSE1456 | GPL96 | Pawitan *et al* | Gene expression of breast cancer tissue in a large population-based cohort of Swedish patients | Breast Cancer Res 2005 |
| GSE45255 | GPL96 | Nagalla *et al* | Expression Profiles of Breast Tumors from Singapore and Europe | Genome Biol 2013 |
| GSE4922 | GPL96 | Ivshina *et al* | Genetic Reclassification of Histologic Grade Delineates New Clinical Subtypes of Breast Cancer | Cancer Res 2006 |
| GSE7390 | GPL96 | Desmedt *et al* | Strong Time Dependence of the 76-Gene Prognostic Signature | Clin Cancer Res 2007 |
| GSE12093 | GPL96 | Zhang *et al* | The 76-gene Signature Defines High-Risk Patients that Benefit from Adjuvant Tamoxifen Therapy | Breast Cancer Res Treat 2009 |
| GSE2034 | GPL96 | Wang *et al* | Breast cancer relapse free survival | Lancet 2005 |
| GSE17705 | GPL96 | Symmans *et al* | Endocrine Sensitivity Index Validation Dataset | J Clin Oncol 2010 |
| GSE25066 | GPL96 | Hatzis *et al* | Genomic predictor of response and survival following neoadjuvant taxane-anthracycline chemotherapy in breast cancer | JAMA 2011 |
| GSE2990 | GPL96 | Sotiriou *et al* | Gene Expression Profiling in Breast Cancer: Understanding the Molecular Basis of Histologic Grade To Improve Prognosis | J Natl Cancer Inst 2006 |
| GSE11121 | GPL96 | Schmidt *et al* | The humoral immune system has a key prognostic impact in node-negative breast cancer | Cancer Res 2008 |

GEO, gene expression omnibus; GSE, GEO datasets number prefixes; GPL570, a type of oligonucleotide gene chip (U133 plus 2.0) from the affymetrix platform. GPL96, a type of oligonucleotide gene chip (U133A) from the affymetrix platform.

**Supplementary Table S2:** Multivariate analysis of age, ER-, PR-status, lymph node, grade and the 92-probe signature in relation to the 5-years survival. (≥5yrs).

| Parameters | HR (95%CI) | *p* Value |
|---|---|---|
| **Analysis without 92 gene signature** | | |
| **Age (years)** | 0.997 (0.963–1.033) | 0.872 |
| **ER status (+/–)** | 0.752 (0.142–3.977) | 0.737 |
| **PR status (+/–)** | 1.567 (0.376–6.522) | 0.537 |
| **Lymph node (yes/no)** | 2.349 (0.978–5.643) | 0.056 |
| **Grade (1, 2, 3)** | 1.462 (0.848–2.520) | 0.172 |
| **Analysis with 92 gene signature** | | |
| **Age (years)** | 1.001 (0.964–1.039) | 0.958 |
| **ER status (+/–)** | 1.999 (0.316–12.643) | 0.462 |
| **PR status (+/–)** | 1.646 (0.381–7.115) | 0.505 |
| **Lymph node (yes/no)** | 2.696 (1.105–6.577) | 0.029 |
| **Grade (1, 2, 3)** | 1.237 (0.703–2.177) | 0.460 |
| **92 gene signature (low/high)** | 4.136 (1.306–13.102) | 0.016 |

The multivariate model included 192 patients for DFS from GSE31448 and GSE9195, owing to the missing values in twenty two. A low risk was defined as a prognostic index less than or equal to -0.272144, and a high risk as a PI higher than -0.272144. HR, hazard ratio; CI, confident interval; ER, estrogen receptor; PR, progesterone receptor.

**Supplementary Table S3:** Summary of the 92 probe sets in prognostic expression signature.

**Supplementary Table S4:** Comparison of genes in the 92-probes with published breast cancer gene signatures.

| Gene symbol | Name | Chang et al 2011 | Prat et al 2013 | Liu et al 2007 | Pawitan et al 2005 | Sparano et al 2007 | Van ‘t Veer et al 2002 |
|---|---|---|---|---|---|---|---|
| ABAT | 4-aminobutyrate aminotransferase | * | | | | | |
| CA12 | carbonic anhydrase XII | * | | | | | |
| CDC20 | cell division cycle 20 | | | | * | | |
| ERBB4 | v-erb-a erythroblastic leukemia viral oncogene homolog 4 (avian) | | | * | | | |
| ESR1 | estrogen receptor 1 | * | * | | | * | |
| FOXC1 | forkhead box C1_Ref4 | | * | | | | |
| GATA3 | GATA binding protein 3 | * | * | | | | |
| HSPA2 | heat shock 70kDa protein 2 | | | * | * | | |
| IGF1 | insulin-like growth factor 1 (somatomedin C)_Ref1 | | | | * | | |
| MAPT | microtubule-associated protein tau | * | * | * | | | |
| PGR | progesterone receptor | | | | | * | |
| RARRES1 | retinoic acid receptor responder (tazarotene induced) 1 | * | | | | | |
| SCUBE2 | signal peptide, CUB domain, EGF-like 2 | | | | | * | |
| SLC39A6 | solute carrier family 39 (zinc transporter), member 6 | * | | | | | |
| SOX11 | SRY (sex determining region Y)-box 11 | | * | | | | |
| TBC1D9 | TBC1 domain family, member 9 (with GRAM domain) | * | | | | | |
